# Looking for alternative treatments for bovine and caprine mastitis: Evaluation of the potential of *Calliandra surinamensis* leaf pinnulae lectin (CasuL), both alone and in combination with antibiotics

**DOI:** 10.1002/mbo3.869

**Published:** 2019-05-17

**Authors:** Thamara F. Procópio, Maiara C. Moura, Elinaldo F. L. Bento, Tatiana Soares, Luana C. B. B. Coelho, Raquel P. Bezerra, Rinaldo A. Mota, Ana Lúcia F. Porto, Patrícia M. G. Paiva, Thiago H. Napoleão

**Affiliations:** ^1^ Departamento de Bioquímica, Centro de Biociências Universidade Federal de Pernambuco Recife Brazil; ^2^ Centro de Tecnologias Estratégicas do Nordeste Recife Brazil; ^3^ Departamento de Morfologia e Fisiologia Animal Universidade Federal Rural de Pernambuco Recife Brazil; ^4^ Departamento de Medicina Veterinária Universidade Federal Rural de Pernambuco Recife Brazil

**Keywords:** antibiofilm activity, bacteriostatic agent, mastitis, *Staphylococcus*, synergism

## Abstract

This work aimed to evaluate the effects of CasuL on growth and viability of 15 mastitis isolates from cows and goats, to determine the synergistic potential between CasuL and antibiotics, and to investigate the effects on bacterial ultrastructure and antibiofilm activity. The lectin inhibited the growth of *Staphylococcus* isolates from either bovine (Ssp6PD and Sa) or caprine (Ssp5D and Ssp01) mastitis. The minimal inhibitory concentrations were ranged from 3.75 to 15 µg/ml. Synergistic effect was observed for CasuL‐tetracycline against Sa and Ssp6PD and CasuL‐ampicillin against Ssp01. No structural damage was observed under the scanning electron microscope in CasuL treatments. Flow cytometry analysis using thiazol orange and propidium iodide demonstrated that CasuL was unable to reduce the cell viability of the isolates tested. At sub‐inhibitory concentrations, CasuL reduced biofilm formation by the isolates Sa and Ssp5D. However, CasuL‐tetracycline and CasuL‐ampicillin combinations inhibited biofilm formation by Ssp6PD and Ssp01, respectively. In conclusion, CasuL is a bacteriostatic and antibiofilm agent against some mastitis isolates and displayed a synergistic potential when used in combination with either ampicillin (against one isolate) or tetracycline (against two isolates). The results stimulate the evaluation of CasuL for the treatment of mastitis, particularly when used in conjunction with antibiotics.

## Graphical abstract

CasuL is a bacteriostatic and antibiofilm agent against mastitis isolates and displayed a synergistic potential when used in combination with either ampicillin or tetracycline.

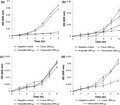

## 1 INTRODUCTION

Mastitis is an infection that is caused by certain microorganisms that are present in the mammary glands. It leads to functional impairment resulting from the destruction of milk‐producing tissues (Mushtaq et al., 2018; Schroeder, 1997). Reduced milk production caused by cases of mastitis in bovines and caprines has an enormous economic impact on the dairy industry (Guimarães et al., 2017). The presence of enterotoxins in milk and the spread of antibiotic‐resistant microorganisms are further problems associated with cases of mastitis (Scali, Camussone, Calvinho, Cipolla, & Zecconi, 2015).

Although fungi, viruses, and algae can cause mastitis, bacteria are responsible for the highest infection rates in the mammary glands of cows and goats (Costa, 1991; Spanamberg, Sanches, Santurio, & Ferreiro, 2009). *Staphylococcus aureus* is one of the main causes of clinical and subclinical mastitis (Klein et al., 2015; Peixoto, França, Souza Júnior, Veschi, & Costa, 2010; Zadoks & Fitzpatrick, 2009). However, other bacteria such as streptococci, *Escherichia coli*, and *Klebsiella pneumoniae* are also responsible for this infection (Contreras & Rodríguez, 2011). Mastitis treatment and prevention consists mainly of the use of antibiotics and proper animal handling to prevent the spread of the disease to healthy animals. However, the use of antibiotics requires caution in order to avoid the emergence of resistant bacteria (Costa et al., 2013; Moritz & Moritz, 2016). Krewer et al. (2013) found simultaneous resistance to three or more antibiotics in 65.6% of *Staphylococcus* isolates that cause mastitis.

Biofilms are complex and structured communities of microorganisms enclosed in a self‐produced polymeric matrix that contains exopolysaccharides, proteins, teichoic acids, enzymes, and extracellular DNA (Klein et al., 2015). Biofilms give these microorganisms protection against environmental adversities and a higher tolerance (10–1,000 times) to antibiotics as compared to planktonic forms (Cerca et al., 2005; Kumar, Alam, Rani, Ehtesham, & Hasnain, 2017). It is believed that biofilm development may contribute to the low efficacy of certain therapies used in bovine mastitis treatment, as well as to the difficulties of treating recurrent infections (Martins et al., 2016; Melchior, Vaarkamp, & Fink‐Gremmels, 2006).

Lectins are proteins of non‐immunological origin that bind specifically and reversibly to free or conjugated carbohydrates. These proteins have significant antibacterial potential which is attributed to their ability to bind molecules present in the surface of gram‐positive and gram‐negative cells, leading to damage to the cell wall, loss of metabolic stability, inhibition of cell growth, and reduction in cell viability (Procópio, Moura, et al., 2017). Lectins can also interfere with adhesion and invasion of host cells by bacteria (Silva et al., 2016). Finally, lectins have been reported to be able to both prevent biofilm formation and eradicate already established biofilms (Moura et al., 2015; Moura, Trentin, et al., 2017).

CasuL is a thermo‐stable and acidic lectin that was previously isolated from the leaf pinnulae of *Calliandra surinamensis* (“pink powder puff,” “pompon du marin,” or “esponjinha‐rosa”). This lectin displayed cytotoxic activity against human cancer cells, fungistatic, and fungicidal effects on *Candida krusei*, and bacteriostatic and antibiofilm properties against human pathogenic bacteria (Procópio, Patriota, et al., 2017).

In view of a previous report on the antibacterial effects of CasuL and the problems associated with bovine and caprine mastitis, this work aimed to evaluate the bacteriostatic and bactericide effects of CasuL on fifteen mastitis isolates, to determine the synergistic potential between CasuL and commercially available antibiotics, and to investigate the effects of CasuL alone or combined with antibiotics on bacterial ultrastructure and antibiofilm activity.

## 2 MATERIALS AND METHODS

### 2.1 Lectin purification

*Calliandra surinamensis* leaves were collected at Recife (Pernambuco, Brazil) and dried for 2 weeks at 28ºC. Plant collection was performed under authorization (36,301) of the *Instituto Chico Mendes de Conservação da Biodiversidade* (ICMBio). The access was recorded (A2E872B) in the *Sistema Nacional de Gestão do Patrimônio Genético e do Conhecimento Tradicional Associado* (SisGen). The pinnulae were then detached and ground using a blender. CasuL was then purified from the pinnulae powder according to the protocol described by Procópio, Patriota, et al. (2017). Briefly, an extract was prepared by suspending 10 g of the powder in 100 ml of 0.15 mol/L NaCl with magnetic stirring for 16 hr, followed by filtration and centrifugation (12,000 g, 15 min, 4ºC). The extract was then treated with ammonium sulfate at 60% saturation (Green & Hughes, 1955) and the precipitated fraction obtained was dialyzed against distilled water (4 hr) and 0.15 mol/L NaCl (4 hr), and then loaded onto a Sephadex G‐75 column (30.0 × 1.0 cm) equilibrated with 0.15 mol/L NaCl. Elution was monitored by absorbance at 280 nm and CasuL was recovered in fractions 9–15. Isolated CasuL was exhaustively dialyzed (6 hr, two liquid changes) against distilled water before use in the antibacterial assays.

### 2.2 Protein concentration

Protein concentration was determined according to Lowry, Rosebrough, Farr, and Randall (1951) using bovine serum albumin (31.25–500 µg/ml) as the standard.

### 2.3 Hemagglutinating activity

The hemagglutinating activity (HA) assay was used to determine the carbohydrate‐binding ability of CasuL. A 2.5% (v/v) suspension of glutaraldehyde‐treated rabbit erythrocytes in 0.15 mol/L NaCl was used. The Ethics Committee on Animal Use of the *Universidade Federal de Pernambuco* approved the method that was used to collect erythrocytes (process 23076.033782/2015‐70). The HA was determined as described by Procópio, Patriota, et al. (2017) and the number of HA units was determined as the reciprocal of the highest dilution of the lectin that was able to agglutinate erythrocytes. Specific HA was calculated by determining the ratio of HA to protein concentration (mg/ml). An HA inhibitory assay was performed by incubating CasuL for 15 min with fetuin prior to the addition of erythrocyte suspension.

### 2.4 Bacterial isolates

Fifteen mastitis bacterial strains isolated from goats and cows (Table 1) were obtained from the collection maintained by the *Laboratório de Tecnologia de Bioativos* of the *Departamento de Morfologia e Fisiologia Animal* from the *Universidade Federal Rural de Pernambuco*. The majority of the isolates tested belonged to the *Staphylococcus* genus (*S.  aureus*, Sa; *Staphylococcus* sp., Ssp). One of the isolates belonged to the *Corynebacterium* genus and one of the isolates was an *Escherichia coli* strain. The stock cultures were maintained at −20ºC in sterile Mueller Hinton Broth (MHB) with 10% (v/v) glycerol. For use in the assays, the bacteria were cultured in Mueller Hinton Agar (MHA) overnight at 37°C and the culture density was adjusted turbidimetrically at 600 nm (OD_600_) to 1 × 10^8^ colony forming units (CFU) per ml in sterile 0.15 mol/L NaCl. This suspension was subsequently diluted in saline solution to 1 × 10^6^ CFU/ml to yield approximately 1 × 10^5^ CFU/ml as the final concentration used in the antibacterial assay described below.

**Table 1:** Mastitis isolates and minimum inhibitory concentration (MIC) values of CasuL, ampicillin and tetracycline

Isolates	MIC (µg/ml)		
	CasuL	Ampicillin	Tetracycline
Bovine mastitis			
CORY (*Corynebacterium* sp.)	ND	ND	0.25
Ec (*Escherichia coli*)	ND	4.00	0.12
Sa (*Staphylococcus aureus*)	3.75	8.00	0.50
Ssp13PD (*Staphylococcus* sp.)	ND	0.12	0.12
Ssp18PE (*Staphylococcus* sp.)	ND	0.50	8.00
Ssp5PE (*Staphylococcus* sp.)	ND	0.12	ND
Ssp6PD (*Staphylococcus* sp.)	3.75	4.00	2.00
Caprine mastitis			
Sa24 (*Staphylococcus aureus*)	ND	8.00	0.50
Ssp01 (*Staphylococcus* sp.)	15.00	0.50	1.00
Ssp02 (*Staphylococcus* sp.)	ND	ND	2.00
Ssp310 (*Staphylococcus* sp.)	ND	8.00	0.12
Ssp48 (*Staphylococcus* sp.)	ND	0.12	ND
Ssp5D (*Staphylococcus* sp.)	3.75	ND	4.00
Ssp601 (*Staphylococcus* sp.)	ND	0.50	ND
Ssp9 (*Staphylococcus* sp.)	ND	0.50	4.00

Abbreviation: ND, not detected.

### 2.5 Determination of minimal inhibitory and bactericidal concentrations

The broth microdilution assay was used to determine minimal inhibitory concentrations (MIC) and minimal bactericidal concentrations (MBC) values. First, a twofold serial dilution of either CasuL (37.50 µg/ml) or antibiotic (8.00 µg/ml) in 80 μl of distilled water was performed in a row of a 96‐well microplate. Distilled water (80 μl) was used in the 100% growth control. Next, 40 μl of MHB and 80 µl of bacterial inoculum (in saline) was added. The final concentrations of CasuL in the wells ranged from 0.03 to 15.00 µg/ml. A sterility control contained only medium was also made. The OD_600_ was measured at time zero and following incubation at 37ºC for 24 hr. The MIC was determined as the lowest sample concentration that was able to promote a reduction of OD_600 _by 50% or higher in comparison with the 100% growth control (Amsterdam, 1996). Each assay was performed in duplicate and three independent experiments were performed.

To determine the MBC, the supernatants from each well containing CasuL at concentration ≥ MIC were smeared onto MHA medium and the plates were then incubated for 24 hr at 37ºC. The MBC corresponded to the lowest sample concentration that was able to reduce the number of CFU in 99.9% in comparison with the initial inoculum.

### 2.6 Synergism assay

Possible synergistic effects between CasuL and antibiotics (ampicillin or tetracycline) were evaluated using the method described by Pillai, Moellering, and Eliopoulos (2005). Lectin‐susceptible isolates (Sa, Ssp6PD, Ssp5D, and Ssp01) were tested in the assays. Each experiment corresponded to two rows of a 96‐well microplate. CasuL was added (80 µl) to the fourth well of the first row and a serial two‐fold dilution in sterile Milli‐Q water was performed until the penultimate well of the second row. Next, the antibiotic was added (80 µl) to the penultimate well of the second row and a two‐fold serial dilution was carried out in the opposite direction until the fourth well of the first row. The third well of the first row contained only CasuL and the last well of the second row contained only the drug. Forty microlitre of MHB was added to all wells, except the first, which contained 200 µl of culture medium and served as a sterility control. The second well corresponded to the 100% growth control. The final concentrations of CasuL in the wells ranged from MIC/262,144 (penultimate well of second row) to 2 × MIC (third well of the first row). The final concentrations of the drug in the wells ranged from MIC/262,144 (fourth well of the first row) to 2 × MIC (last well of the second row). Each well, except the first, was inoculated with microbial suspension (80 µl at 10^5 ^CFU/ml) and incubated at 37ºC. The experiment was monitored by measuring the OD_600 _at time zero and after 24 hr. An evaluation of the interaction between the different treatments was performed by determining the fractional inhibitory concentration index (FICI), as follows: FICI = (MIC of CasuL in combination/MIC of CasuL alone) + (MIC of antibiotic in combination/MIC of antibiotic alone). The combinations were classified as synergistic (FICI ≤ 0.5), additive (0.5 < FICI ≤ 1), indifferent (1 < FICI ≤ 2), or antagonistic (FICI > 2).

### 2.7 Growth curves

Six‐hour growth curves were determined for CasuL‐sensitive isolates using either the lectin alone, or synergic combinations of CasuL with antibiotics. This assay was performed in 96‐well microtiter plates according to Gaidamashvili and Van Staden (2002). Eighty microlitre of the inoculums (10^6 ^CFU/ml) in the exponential growth phase were incubated with 40 µl of MHB and 80 µl of the lectin, ampicillin, or tetracycline (at MIC), or with a synergic combination. In the 100% growth control, sterile distilled water (negative control) was used instead of CasuL. The plates were incubated at 37ºC and the OD_600 _was measured every hour.

### 2.8 Scanning electron microscopy

Three‐dimensional images of bacterial cells were obtained by Scanning electron microscopy (*SEM*). The cells (1.2 ml; 10^6^ CFU/ml) were incubated with MHB (0.6 ml) and 400 µl of CasuL (at MIC), CasuL‐antibiotic synergic combination, or distilled water (negative control). After incubation (24 hr at 37°C), the samples were centrifuged (300 g; 10 min, 25°C) and the cell pellet was washed three times with 0.1 mol/L phosphate‐buffered saline (PBS) pH 7.0, followed by three washings in 0.1 mol/L cacodylate buffer, and then fixation in 2.5% glutaraldehyde/4% paraformaldehyde/5 mmol/l CaCl_2_ in 0.1 mol/L cacodylate buffer pH 7.2 for 30 min at 28ºC. The cells were then allowed to adhere to stubs (Ø 12.7 mm, 9 mm length; Ted Pella Inc., Redding, CA) and postfixed for 1 hr with 1% osmium tetroxide/0.8% potassium ferricyanide/5 mmol/L CaCl_2_ in 0.1 mol/L cacodylate buffer, pH 7.2. The cells were dehydrated in graded acetone, critical‐point‐dried with CO_2_, coated with a 20 nm‐thick gold layer, and observed with a Quanta 200F (FEI Company, Hilsboro, OR) scanning electron microscope.

### 2.9 Cell viability analysis

The viability of bacterial cells treated with CasuL was evaluated using the Cell Viability Kit of BD Biosciences (San Jose, CA). The isolates were incubated with the lectin at the MIC as described above. The negative control was prepared by adding distilled water instead of CasuL. For the positive control, cells were treated with 70% (v/v) isopropyl alcohol for 1 hr. Following incubation (24 hr, 37°C), the samples were centrifuged (300 g, 10 min, 25°C) and the cell pellets were washed three times with 0.1 mol/L PBS pH 7.0. Next, 42 µmol/L thiazole orange (5 µl) and 4.3 mmol/L propidium iodide (5 µl) was added to the assays, which was vortexed and incubated for 5 min at 25ºC. Next, 50 μl of a fluorescent bead suspension (BD Liquid Counting Beads) was added, and the mixture was vortexed for 30 s. Data acquisition was performed in a BD Accuri C6 cytometer (BD Biosciences) with an SSC threshold of 200 and stopped after gating 20,000 events for each sample. Analysis was performed in the BD Accuri C6 Software.

### 2.10 Antibiofilm assay

Forty microlitre of MHB medium, 80 µl of ultrapure Milli‐Q water (negative control) or 80 µl of CasuL (1/8, 1/4, 1/2, and 1 × MIC, in Milli‐Q water), and 80 µl of the bacterial suspension (10^8^ CFU/ml in saline) were added to each well of a 96‐well polystyrene microplate. The OD_600_ was recorded at this time and the microplate was further incubated at 37°C for 24 hr. After this period, the OD_600_ was read again, the content of the wells was removed, and the plate wells were washed three times with saline solution. The remaining attached cells were heat‐fixed at 50°C for 60 min. They were then fixed with absolute methanol for 30 min and stained with 0.4% (w/v) crystal violet for 25 min at 25°C. After washing with water, the stain bound to the biofilm was solubilized with absolute ethanol (25‐min incubation) and the absorbance was measured at 570 nm (Trentin et al., 2011). Tetracycline and/or ampicillin (1/8, 1/4, 1/2, and 1 × MIC, in Milli‐Q water) was used as positive control. In addition, the synergic combinations (regarding bacteriostatic effects) were evaluated for their antibiofilm effect. Three independent experiments were performed in triplicate.

### 2.11 Statistical analysis

The data were expressed as the mean or the percent mean ± *SD* and statistical differences were determined using Tukey’s test. A *p* < 0.05 was considered to be statistically significant.

## 3 RESULTS

CasuL was isolated according to the protocol previously established by Procópio, Patriota, et al. (2017). The isolated lectin showed a specific HA of 1,420.0 and was inhibited by fetuin, as in the previous report, confirming that the carbohydrate‐binding activity of the sample was effective. CasuL was able to inhibit the growth of four isolates. The MIC values are presented in Table 1 and ranged from 3.75 to 15.0 µg/ml. It was not possible to determine the MBC as none of the concentrations tested prevented bacterial growth in agar. The MIC values for the reference drugs (ampicillin and tetracycline) are also shown in Table 1. Eight isolates were found to lack sensitivity to at least one of the antibiotics.

The potential synergy between CasuL and antibiotics was evaluated, and the results are shown in Tables 2 and 3. A synergistic effect was observed for the combination CasuL‐ampicillin against the isolate Ssp01 (Table 2) and for the combination CasuL‐tetracycline against the isolates Sa and Ssp6PD (Table 3). An additive effect was observed against the isolate Ssp5D, while antagonism was observed for the combinations CasuL‐ampicillin and CasuL‐tetracycline, against the strains Sa and Ssp01, respectively.

**Table 2:** Evaluation of antimicrobial activity of CasuL in combination with ampicillin against *Staphylococcus* isolates from bovine and caprine mastitis

Isolate	MIC (µg/ml)				FICI	Effect
	Alone		In CasuL + ampicillin combination			
	CasuL	Ampicillin	CasuL^a^	Ampicillin^a^		
Sa (*Staphylococcus aureus*)	3.75	8.00	ND	ND	NC	Antagonistic
Ssp6PD (*Staphylococcus* sp.)	3.75	4.00	3.00 × 10^−5^	8.00	2.00	Indifferent
Ssp01 (*Staphylococcus* sp.)	15.00	0.50	1.83 × 10^−3^	1.56 × 10^−2^	0.03	Synergistic

Note

Classification: synergistic (FICI ≤ 0.5), additive (0.5 < FICI ≤ 1), indifferent (1 < FICI ≤ 2) or antagonistic (FICI > 2).

Abbreviations: FICI, fractional inhibitory concentration index; MIC, minimum inhibitory concentration; NC, not calculated; ND, not detected.

a The values correspond to the concentration of CasuL or ampicillin in the microplate well containing both compounds.

**Table 3:** Evaluation of antimicrobial activity of CasuL in combination with tetracycline against *Staphylococcus* isolates from bovine and caprine mastitis

Isolate	MIC (µg/ml)				FICI		Effect	
	Alone		In CasuL + tetracycline combination					
	CasuL	Tetracycline	CasuL^a^	Tetracycline^a^				
Sa (*Staphylococcus aureus*)	3.75	0.50	2.30 × 10^−4^	0.12		0.250		Synergistic	
Ssp6PD (*Staphylococcus* sp.)	3.75	2.00	3.66 × 10^−3^	3.12 × 10^−2^		0.016		Synergistic	
Ssp01 (*Staphylococcus* sp.)	15.00	1.00	ND	ND		NC		Antagonistic	
Ssp5D (*Staphylococcus* sp.)	3.75	4.00	6.00 × 10^−5^	4.0		1.000		Additive	

Note

Classification: synergistic (FICI ≤ 0.5), additive (0.5 < FICI ≤ 1), indifferent (1 < FICI ≤ 2) or antagonistic (FICI > 2).

Abbreviations: FICI, fractional inhibitory concentration index; MIC, minimum inhibitory concentration; NC, not calculated; ND, not detected.

a The values correspond to the concentration of CasuL or ampicillin in the microplate well containing both compounds.

**Figure 1:**
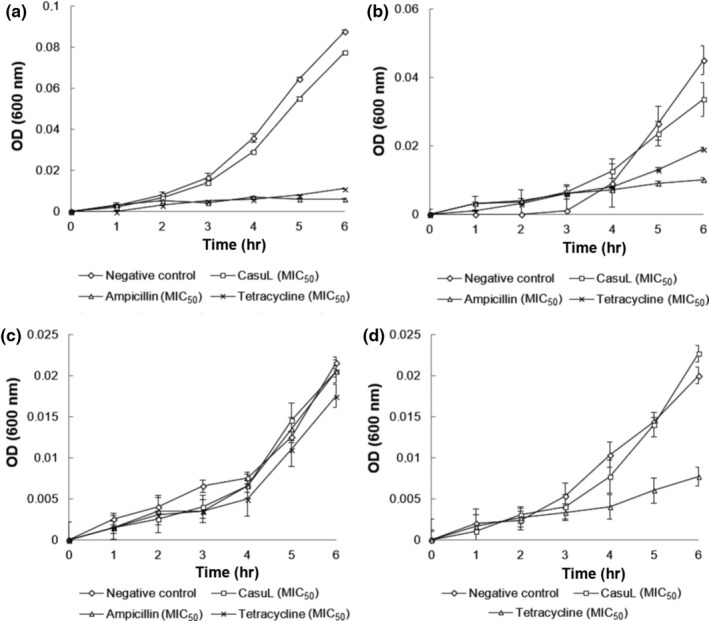
Growth curves of the mastitis isolates Sa (a), Ssp6PD (b), Ssp01 (c), and Ssp5D (d) in absence or presence of CasuL, ampicillin, or tetracycline at their respective minimal inhibitory concentrations (MIC). The optical density (OD) at 600 nm was determined every hour for a period of 6 hr. For the negative control, cells were treated with distilled water instead of antibacterial agent. Data are expressed as the mean ± *SD*. All the MIC values can be seen in Table 1

Six‐hour growth curves were determined for the lectin‐sensitive isolates in the absence and presence of either CasuL or antibiotics (Figure 1). When incubated with the lectin, all of the isolates grew similar to the negative control (100% growth), indicating that the bacteriostatic effect only appears later on (after the 6‐hr period evaluated). The presence of tetracycline and ampicillin led to a reduction in the growth of the Sa isolate at 3 hr of incubation (Figure 1a), while the isolate Ssp6PD had its growth reduced after 5 hr of incubation with both antibiotics (Figure 1b). For the isolate Ssp01, neither antibiotic showed any inhibitory effect in the first 6 hr of incubation (Figure 1c), similar to CasuL. Finally, for the isolate Ssp5D, tetracycline was shown to be able to inhibit growth from the fourth hour of incubation on ward (Figure 1d). This isolate was not sensitive to ampicillin.

**Figure 2:**
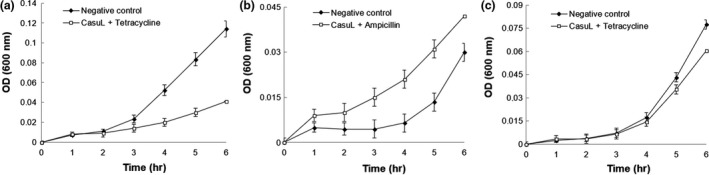
Growth curves of the mastitis isolates Sa (a), Ssp01 (b), and Ssp6PD (c) in absence or presence of CasuL‐tetracyline (a,c) or CasuL‐ampicillin (b) synergic combinations. The optical density (OD) at 600 nm was determined every hour for a period of 6 hr. For the negative control, cells were treated with distilled water instead of antibacterial agent. Data are expressed as the mean ± *SD*. The concentration of CasuL and antibiotics in the synergic combinations can be seen in Table 2

Growth curves for synergistic combinations of CasuL and antibiotics were also determined in order to analyze whether the time–effect relationship of these antibacterial agents could be improved when combined. Figure 2a shows that the growth of isolate Sa was reduced by treatment with the CasuL‐tetracycline combination for 4 hr. For the isolates Ssp01 (Figure 2b) and Ssp6PD (Figure 2c), treatment with the combinations CasuL‐ampicillin and CasuL‐tetracycline did not result in an inhibition of growth within short incubation periods.

**Figure 3:**
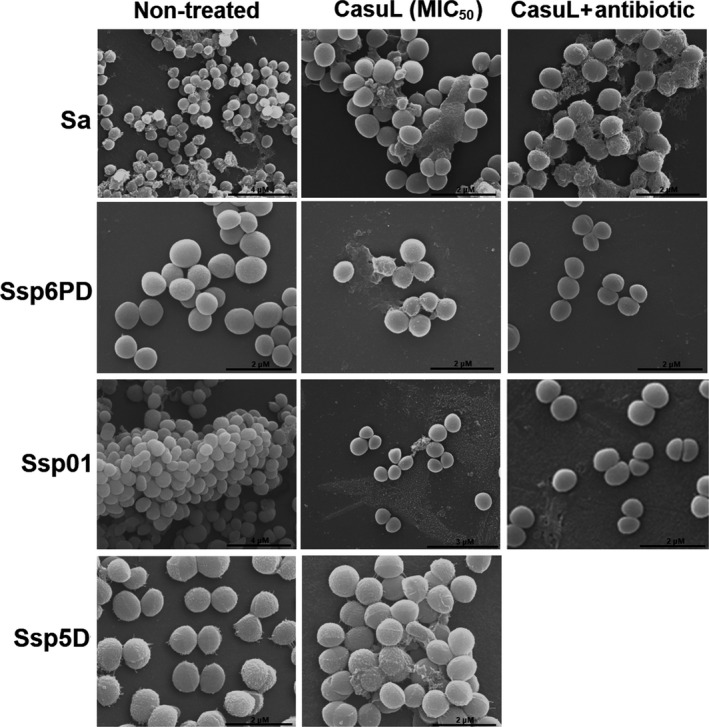
Scanning electron microscopy of bacterial cells of the isolates Sa, Ssp6PD, Ssp01, and Ssp5D following exposure to either CasuL at the minimal inhibitory concentration (MIC),or to CasuL‐antibiotic synergic combination (except for Ssp5D isolate). For the negative control, cells were treated with distilled water instead of antibacterial agent. The synergistic combinations used were as follows: CasuL‐tetracycline for isolates Sa and Ssp6PD, and CasuL‐ampicillin for the isolate, Ssp01. Reduction in cell number and cells under incomplete division can be seen in CasuL treatments, but no bacterial surface alteration was observed following treatments with either lectin or with synergic combinations. The MIC values of CasuL can be seen in Table 1. The concentrations of CasuL and antibiotics in the synergic combinations can be seen in Table 2

To determine whether CasuL acts by disrupting the integrity of bacterial cell surface, the isolates were incubated with the lectin at either the respective MIC, or with synergic combinations of CasuL with either tetracycline or ampicillin. The cells were then visualized by *SEM*. Figure 3 shows a reduction in cell number and cells under incomplete division following treatment with CasuL. However, no bacterial surface alteration was observed following treatment with either CasuL or with the CasuL‐antibiotic combinations in comparison with the negative control.

**Figure 4:**
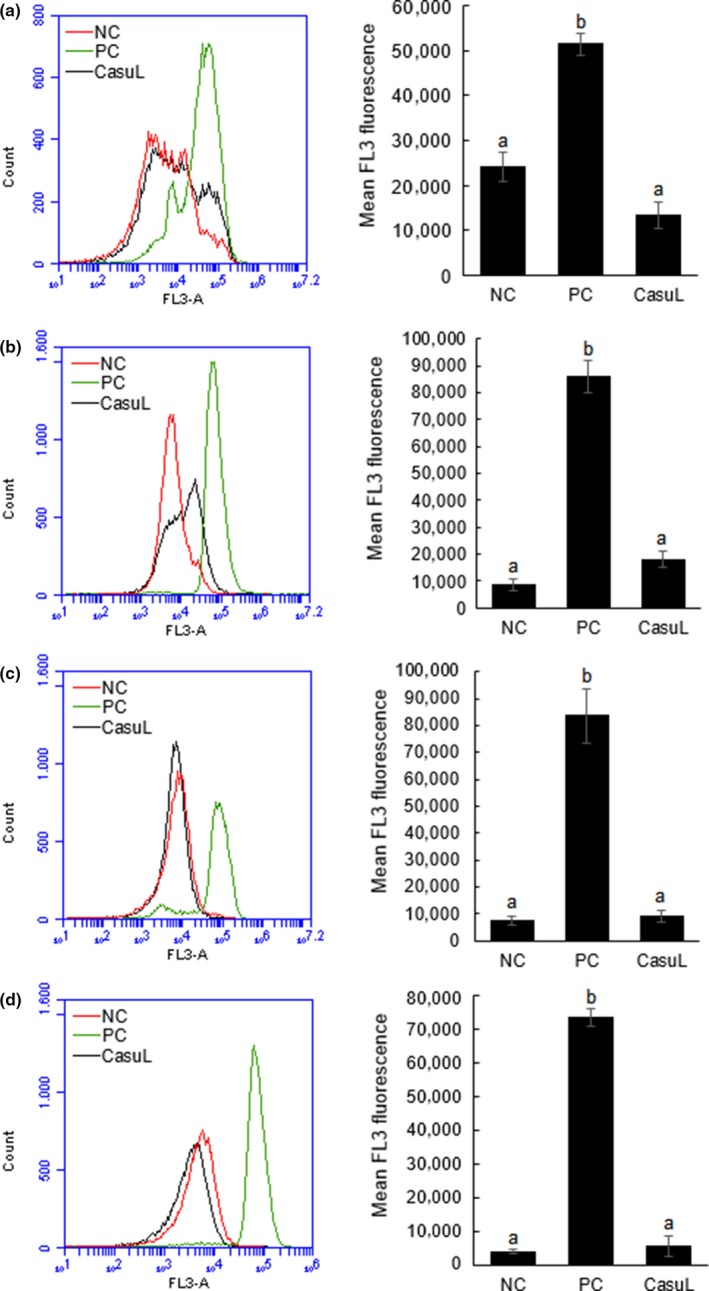
Analysis of cell viability of the isolates Ssp01 (a), Ssp5D (b), Ssp6PD (c), and Sa (d) in absence or presence of CasuL at the minimal inhibitory concentration (MIC) by flow cytometry. Cells incubated in absence of lectin corresponded to the negative control (NC). Isopropyl alcohol (70%, v/v) was used as positive control (PC). Overlay histograms (at left) shows the distribution of unviable cells stained with propidium iodide (FL3 channel) in NC, PC and CasuL groups. Bar charts (at right) display the mean fluorescence in FL3 channel. Data are expressed as the mean ± * SD*. All the MIC values are given in Table 1

The results of *SEM* prompted us to evaluate whether CasuL, in spite of the absence of structural damage, could be affecting bacterial viability. Flow cytometry analysis was performed using the dye thiazol orange to stain all bacterial cells and propidium iodide to stain nonviable cells. The results demonstrate that CasuL did not reduce the cell viability of all the four isolates. The mean fluorescence of propidium iodide (FL3 channel) of lectin‐treated cells was similar to that of the negative control while cells incubated with positive control (isopropyl alcohol) were intensely stained by this probe (Figure 4).

**Figure 5:**
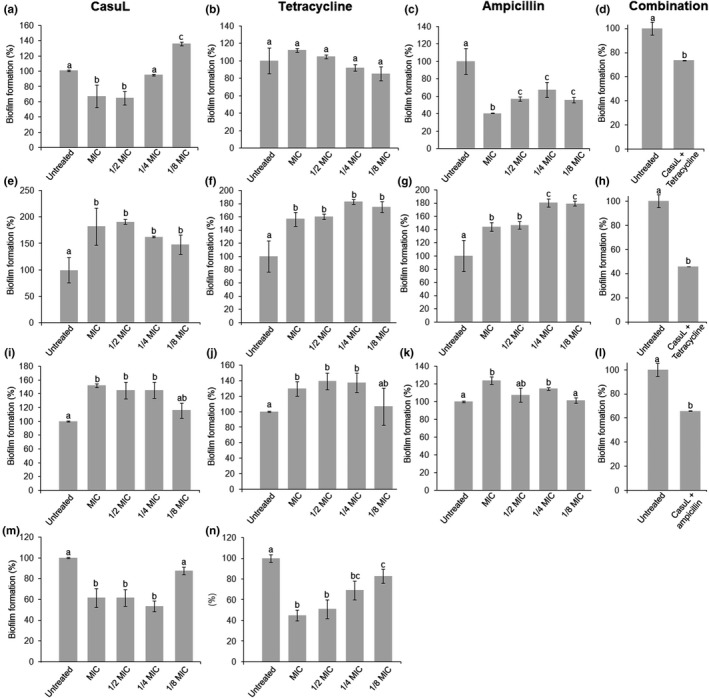
Evaluation of the antibiofilm effect against the isolates Sa (a–d), Ssp6PD (e–h), Ssp01 (i–l), and Ssp5D (m,n) of CasuL(a,e,i,m), tetracycline (b,f,j,n) or ampicillin (c,g,k), all at sub‐inhibitory concentrations, as well as of CasuL‐tetracycline (d,h), and CasuL‐ampicillin (l) combinations. Different letters indicate significant differences (*p* < 0.05) between the treatments and the negative control. The minimal inhibitory concentrations (MIC) of CasuL and antibiotics are given in Table 1. The concentrations of CasuL and antibiotics in the combinations are given in Table 2

Biofilm formation by the Sa isolate was reduced by approximately 30.0% by treatment with CasuL at 1/2 × MIC and MIC (Figure 5a) and by up to 60.0% following treatment with ampicillin (Figure 5c) but was not affected by treatment with tetracycline (Figure 5b).Treatment with the synergic combination CasuL‐tetracycline led to a 26.0% decrease in biofilm development (Figure 5d).

Treatment with CasuL led to an increase in biofilm development by isolate Ssp6PD at all of the concentrations tested (Figure 5e). Neither of the commercial antibiotics was found to act as antibiofilm compounds against Ssp6PD, also, stimulating biofilm formation (Figure 5f,g). Interestingly, the combination, CasuL‐tetracycline, inhibited biofilm formation by almost 60.0% (Figure 5h).

Antibiofilm activity was not observed following treatment of the isolate Ssp01 with CasuL (Figure 5i). While tetracycline was found to stimulate biofilm development (Figure 5j), ampicillin had no effect (Figure 5k). Similar to the result observed for the isolate Ssp6PD, the CasuL‐ampicillin combination showed an antibiofilm effect, inhibiting formation by approximately 35.0% (Figure 5l). CasuL also reduced biofilm formation by isolate Ssp5D from 1/4 × MIC (Figure 5m) and tetracycline reduced biofilm formation at all of the concentrations tested (Figure 5n).

## 4 DISCUSSION

Mastitis is the most frequent type of inflammation that occurs in milk‐producing animals and the disease that has the greatest impact on dairy farming (Vliegher, Fox, Piepers, McDougall, & Barkema, 2012). *S.  aureus* is the main species of bacteria that causes mastitis and its pathogenesis is attributed to a combination of extracellular and virulence factors and properties such the ability to form biofilms and to develop resistance to antibiotics (Cramton, Gerke, Schnell, Nichols, & Götz, 1999; Moormeier & Bayles, 2017; Vancraeynest, Hermans, & Haesebrouck, 2004). A previous report on the bacteriostatic and antibiofilm activities of CasuL on human pathogenic *Staphylococcus saprophyticus* and *S. aureus* isolates (Procópio, Patriota, et al., 2017) encouraged us to evaluate whether this lectin could exert antibacterial effects on *Staphylococcus* and other bacterial isolates from bovine and caprine mastitis.

Combining antimicrobial phytochemicals with commercial drugs expands the field for the application of these natural compounds and can minimize the impact of pathogen resistance (Lewis & Ausubel, 2006; Mushtaq et al., 2018). Interestingly, synergy between CasuL and tetracycline or ampicillin was observed against some isolates. The growth of isolate Sa was reduced by treatment with the CasuL‐tetracycline synergistic combinations after 4 hr. This result demonstrates that the combination was able to affect the bacterial cells within a short incubation period, similar to that was observed for the antibiotic alone, though at a concentration four times greater.

The antibacterial activity of lectins has previously been associated with their ability to bind to peptidoglycans, lipopolysaccharides, and other molecules present in the cell wall, and by interfering with cell growth and viability and promoting structural damage (Iordache, Ionita, Mitrea, Fafaneata, & Pop, 2015; Procópio, Moura, et al., 2017). However, neither CasuL alone nor the synergistic combinations caused structural alterations, which agree with a predominant bacteriostatic effect.

Indeed, flow cytometry showed that CasuL did not reduce the cell viability of all four isolates reinforcing that CasuL probably acts by inhibiting the replication of bacterial cells without killing them. Bacteriostatic agents and bactericidal drugs are both relevant in many clinical situations and can have both disadvantages and advantages depending on the particular case. For example, the lytic action of bactericidal agents may result in an endotoxin surge and the production of a large number of cell wall fragments leading to exacerbated inflammatory reaction. On the other hand, the use of bacteriostatic drugs can minimize the risk of exotoxin‐related shock‐syndrome. An example of this is the effective use of clindamycin to treat *S. aureus* infections (Pankey & Sabath, 2004).

Biofilm‐forming bacteria are usually highly tolerant to conventional antibiotics and are often resistant to the host immune response (Lebeaux, Ghigo, & Beloin, 2014). Recurrent mastitis infections are often attributed to biofilm growth (Melchior et al., 2006). Therefore, we evaluated the antibiofilm activity of CasuL alone at inhibitory and sub‐inhibitory concentrations as well as the antibiofilm activity of CasuL‐antibiotic combinations. CasuL was found to be less effective than a C‐type lectin from *Bothrops jararacussu* (3.12–100.0 µg/ml), which inhibited biofilm formation by a *S.  aureus* isolate from bovine mastitis by over 50.0% (Klein et al., 2015).

When used alone, CasuL and commercial antibiotics led to an increase in biofilm development by isolates Ssp6PD and Ssp01. It is believed that biofilm formation can be used as a defensive strategy by bacteria to escape the effects of antimicrobial agents (Moura, Napoleão, Paiva, & Coelho, 2017). On the other hand, the combinations CasuL‐tetracycline and CasuL‐ampicillin showed antibiofilm effect. These results are interesting as treatment with these combinations not only reduced the amount of CasuL and antibiotic required to inhibit bacterial growth, but also neutralized the biofilm stimulatory effect of both the antimicrobial agents. A synergistic effect in antibiofilm activity was also detected for the antimicrobial peptide coprisin when it was applied in combination with the antibiotics ampicillin, vancomycin, or chloramphenicol (Hwang et al., 2013).

In conclusion, CasuL displayed bacteriostatic activity against four mastitis isolates from the *Staphylococcus* genus as well as synergistic potential against three isolates when given in combination with either ampicillin or tetracycline. The antibacterial activity of CasuL does not cause structural damage or impairment of cell viability. This correlates with the absence of bactericidal action and defines it as a bacteriostatic drug. At sub‐inhibitory concentrations, CasuL acted as an antibiofilm agent against *S. aureus* and one *Staphylococcus* sp. isolate. It is important to highlight that two lectin‐sensitive isolates displayed an ability to respond to the presence of an antibacterial compound by forming biofilm. However, the CasuL‐antibiotic combinations were able to prevent this response. The results of our work suggest that it would be worthwhile to carry to further studies to evaluate the in vivo effects of CasuL for the treatment of some cases of mastitis, since this lectin does not show a broad spectrum of action. Before this, studies on the toxicity of CasuL to animals should be performed.

## CONFLICT OF INTERESTS

The authors declare no conflict of interest.

## AUTHOR CONTRIBUTIONS

TFP, MCM, ALFP, THN conceptualized the study. TFP, MCM, TS, THN involved in formal analysis. LCBBC, RPB, RAM, ALFP, PMGP, THN obtained funding acquisition. TFP, MCM, EFLB, TS involved in investigation. TFP, MCM, EFLC, TS, THN performed the experimental methodology. THN involved in project administration. LCBBC, RPB, RAM, ALFP, PMGP, THN obtained the resources. TFP, MCM, THN visualized the results obtained. TFP, MCM, THN written the original draft. ALFP, THN involved in writing, reviewing and editing the draft.

## ETHICS STATEMENT

None required.

## Data Availability

All data generated or analyzed during this study are included in this published article. Raw data are available from the corresponding author on reasonable request.
